# Osiris: accessible and reproducible phylogenetic and phylogenomic analyses within the Galaxy workflow management system

**DOI:** 10.1186/1471-2105-15-230

**Published:** 2014-07-02

**Authors:** Todd H Oakley, Markos A Alexandrou, Roger Ngo, M Sabrina Pankey, Celia K C Churchill, William Chen, Karl B Lopker

**Affiliations:** 1 Ecology, Evolution, and Marine Biology, University of California-Santa Barbara, Santa Barbara, CA 93106, USA

**Keywords:** Phylogenomics, Phylogenetics, Galaxy, Orthology, Assembly, Next-generation sequence analysis, Sequence alignment, Tree estimation

## Abstract

**Background:**

Phylogenetic tools and ‘tree-thinking’ approaches increasingly permeate all biological research. At the same time, phylogenetic data sets are expanding at breakneck pace, facilitated by increasingly economical sequencing technologies. Therefore, there is an urgent need for accessible, modular, and sharable tools for phylogenetic analysis.

**Results:**

We developed a suite of wrappers for new and existing phylogenetics tools for the Galaxy workflow management system that we call Osiris. Osiris and Galaxy provide a sharable, standardized, modular user interface, and the ability to easily create complex workflows using a graphical interface. Osiris enables all aspects of phylogenetic analysis within Galaxy, including de novo assembly of high throughput sequencing reads, ortholog identification, multiple sequence alignment, concatenation, phylogenetic tree estimation, and post-tree comparative analysis. The open source files are available on in the Bitbucket public repository and many of the tools are demonstrated on a public web server (http://galaxy-dev.cnsi.ucsb.edu/osiris/).

**Conclusions:**

Osiris can serve as a foundation for other phylogenomic and phylogenetic tool development within the Galaxy platform.

## Background

As phylogenetic data sets expand in scope, especially by leveraging next-generation sequencing technologies, there is an increased need for accessible, reproducible, and transparent computational analyses. Although new analysis paradigms are available, like BEAST XML [1], phyloXML [2], NeXML [3] (and others), reproducibility of phylogenetic analyses is still hampered by a lack of standardization of analytical programs, which have varied authors, different requirements, and use multiple file formats. Most programs still use file formats optimized for single-partition data sets. This often results in the construction of local, inaccessible analytical pipelines that are difficult to share and augment. These difficulties are not unique to phylogenetics; they apply to all bioinformatic analyses. Some recent software platforms like MEGA5 [4], Geneious [5], and CLC Genomics Workbench 7.0.3 (http://www.clcbio.com) (CLC Bio, Aarhus, Denmark) are very user friendly and integrative, but they are not fully open-source, which constrains flexibility and future development. A flexible approach to improving transparency in bioinformatics is to employ open source workflow management systems, such as Kepler, GeneProf, Taverna, Armadillo, Galaxy, and others [6-10].

The Galaxy workflow management system has extensive bioinformatic analysis tools, and provides a number of useful features that can be leveraged for phylogenetic analyses. Galaxy is an open source, lightweight system that can incorporate most existing bioinformatics tools. Galaxy works within a web browser and mainly uses a Graphical User Interface, which many phylogeneticists prefer, as evidenced by the popularity of MEGA [4], MacClade [11] and Mesquite [12]. Galaxy already has a large and growing community of users and contributors, and extensive documentation in a wiki, many screen casts, and email lists (galaxyproject.org). At the heart of Galaxy are histories, which track all analyses, and which can be shared easily with other users. Galaxy also allows the construction of sharable workflows and the construction of “pages,” which document the multiple datasets, tools, and histories used for a project such as a publication (http://tinyurl.com/9232vfr). Galaxy already has extensive tools for analyzing next-generation sequencing data (publicly available on the Galaxy Tool Shed: http://toolshed.g2.bx.psu.edu), which is becoming the standard for molecular phylogenetic analysis. Galaxy can easily leverage computer clusters, which are becoming increasingly necessary as phylogenetic datasets expand, and cloud-based computing, which is rapidly increasing in popularity for academic purposes. Despite the appropriateness of Galaxy for phylogenetic analysis, few tools have yet been developed in Galaxy for this purpose.

## Implementation

Here we describe our development of a suite of tools we collectively refer to as Osiris, which allow extensive phylogenetic analyses within the Galaxy bioinformatics platform. We used Perl, Python, and Bash to develop wrappers for many existing programs and for new custom scripts for all steps of phylogenetic analyses, including data download (e.g. PhyLoTA, GenBank and MorphoBank), ortholog determination, sequence alignment, concatenation and file format conversion, phylogeny estimation, tree manipulation and visualization, and post-tree analyses (Table 1). We have focused on the ability to easily use these tools in parallel, analyzing simultaneously multiple genes or data partitions by using a simple tabular data input format we call phytab. The tools are hosted on the web-based hosting service Bitbucket (https://bitbucket.org/osiris_phylogenetics/), which provides revision control: updates can be easily “pushed” to end-users. This approach can be combined with Galaxy’s existing and expanding tool kit, especially sequence assembly and data manipulation tools, and will serve as a foundation for community contributions of other phylogenetics tools in Galaxy. Having an open resource for phylogenetic tools and analyses will improve accessibility, reproducibility, and transparency in the age of increasingly large phylogenetic data sets and complex analyses.

**Table 1:** New tool wrappers developed thus far for phylogenetic analyses in Galaxy

**Analysis stage**	**Tool**	**Ref.**	**Notes**
Get data	Get GB	*	Grab Genbank data from a text list of accession numbers
	Get GB sp	*	Grab all GenBank data from a text list of species
	PhyLoTA with TaxID	*	Pull all genetic data from PhyLoTA using a GenBank Taxonomy ID
	Generate from PhyLoTA	*	Pull phylogenies and genetic data from PhyLoTA with species list
	GenBank strip	*	Extracts sequences from GenBank files by gene name
	GB gene summary	*	Summarizes gene names in a GenBank flatfile
	Get Sequences	*	Creates a file of selected sequences
Orthologs	EvolMap	[13]	Uses species tree and gene distances to determine orthologs and paralogs
	HaMStR	[14]	Pulls orthologous genes from an input file based on HMM gene models
	HMMbuild	[15,16]	Constructs Hidden Markov Models from aligned sequences
	HMMsearch	[15,16]	Searches for similar genes using HMM models
Alignment	Phytab-MUSCLE	[17]	Implements MUSCLE multiple sequence alignment for multiple gene families in parallel
	Phytab-PRANK	[18]	Implements PRANK phylogeny aware multiple sequence alignment
	Mview	[19]	Converts an aligned sequences file in fasta format to html for visualization
	Phytab-MAFFT	[20]	Implements MUSCLE multiple sequence alignment for multiple gene families in parallel
	Alicut and Aliscore	[21]	Implements Alicut and Aliscore to prune ambiguous alignments for multiple gene families in parallel
	Gblocks	[22]	Implements gblocks to prune ambiguous alignments
	Phytab- Similar Sequence Remover	*	Removes percentage of similar sequences using Phytab input
	Sequence Gap Remover	*	Removes gaps from columns of an aligned phylip file
	Trimming Sites	[13]	Allows user to delete sites from an alignment based on percentage threshold
	Phylocatenator	*	Concatenates phytab datasets based on user-specified criteria and writes phylipE format. Also produces partition file for RAxML
	Fasconcat	[23]	Concatenates input sequence files using Phylip, Clustal or FASTA input
Phyloconversion	tnt2table	*	Converts TNT file format from Morphobank into phytab format
	fasta2phylipE	*	Converts fasta format to phylipE format
	Beautifyfasta	*	Converts fasta interleaved format to sequential
	Addstring2fashead	*	Converts fasta file with sequences from same species and gene family to phytab format
	Length Outliers	*	Identifies sequences shorter than average in FASTA file
	Vert_tree_format	[24]	Convert between phylogenetic tree file formats
	Prune Phytab using list	*	Filters Phytab dataset based on user provided list
	Removes Phytab dupes	*	Finds duplicates in Phytab file
Phylogenies	RAxML	[25]	Implements maximum likelihood (ML) search for optimal phylogeny
	Phytab-RAxML-Parsimony	[25]	Searches for MP phylogeny of multiple data partitions simultaneously
	Phytab-RAxML	[25]	Searches for ML phylogeny of multiple data partitions simultaneously
	Phytab-RAxML using starting trees	[25]	Optimizes branch lengths on a starting tree. Multiple partitions simultaneously
	BEAST	[1]	Executes xml for Bayesian phylogenetic analysis
	RAxML-Place Fossil	[25,26]	Finds fossil position on a tree using morphological data and input phylogeny
	NJst	[27]	Produces species tree from input of multiple gene trees
	RAxML Place reads	[26]	Uses RAxML to place sequence reads onto an existing phylogeny
	RAxML Parsimony	[25]	Uses RAxML to calculate a parsimony tree
	Phytab clearcut	[28]	Generate Neighbor Joining phylogeny. Input can be FASTA or Phytab format
	ProtTest	[29]	Selection of best-fit models of protein evolution
	jModelTest	[30]	Selection of best-fit models of nucleotide evolution
	tab2trees	*	Produces phylogeny graphics, one tree per page, from multiple data partitions or data sets
Phylographics	PDpairs	*	Calculates phylogenetic distances for pairs of species on a phylogeny
Phylostatistics	Phytab LB pruner		Identify genes on very long branches
	Long Branch Finder	*	Identifies terminal branches on multiple gene trees which exceed a threshold
	Phylomatic	[31]	Implements phylomatic program
	Tree Support	[24]	Calculates support for nodes of a single tree (bootstrap) using a file of multiple trees
	Branch Attachment Frequency	[24]	Identifies lineage movement in a set of trees
	Leaf Stability	[24]	Reports leaf stability indices for taxa in tree/trees
	TreeAnnotator	[1]	Calculates summary statistics from posterior distribution of bayesian trees
	Prune Taxa	[24]	Removing taxa from a tree or multiple trees
	Thinning Trees	[24]	Sub-sample trees from a posterior distribution
	SHtest	[25]	Uses RAxML to compute an SHtest to compare trees

*Tools developed for Osiris.

## Results and discussion

### A set of tools for reproducible and accessible phylogenetic analyses

#### Tabular file formats

A fundamental innovation of Osiris is the use of tabular (tab-delimited) data formats, which permit highly parallel analyses, retain more information about the data, and add to the flexibility of analyses. Galaxy already makes extensive use of tabular files, which provide a number of advantages, especially for multi-gene, multi-partition phylogenetic analyses that are now the norm in phylogenetics. First, users can easily edit, view and share these files outside Galaxy, in standard text editors, spreadsheet programs, or relational databases. Second, tabular files can clearly store information of different categories important for phylogenetic analyses. In particular, our tools utilize a four-column format we call phytab format, which stores 1) species name, 2) data partition name (such as gene family name), 3) unique id (such as a GenBank accession), and 4) sequence or morphological character data. This allows for flexibility in using the same data set to concatenate data partitions into a ‘supermatrix’, to analyze genes separately and infer a species phylogeny from separate gene phylogenies, or to estimate the phylogeny of gene families themselves, common in developmental biology and molecular evolutionary biology. Equally important, phytab format facilitates parallelization: each gene family can be analyzed on different processors, to accelerate rapid multiple sequence alignment and gene tree estimation.

#### Osiris tool repositories

Tools within the Osiris phylogenetics platform are organized by type in seven directories within one Bitbucket repository: Get Data, Orthologs, Alignment, Phyloconversion, Phylogenies, Phylographics, and Phylostatistics. These directories comprise centralized, version-controlled tool storage on Bitbucket. A phylogenetic analysis using Osiris combines tools in these repository categories with existing bioinformatics tools in Galaxy.

#### Get Data from Online Databases (Getdata repository)

One of the major difficulties in generating large datasets from public databases such as GenBank is the time-consuming process of searching for each species separately, downloading genes individually, and formatting the data for use in downstream applications. We have developed a number of tools that allow the user to download data directly from GenBank or PhyLoTA using species lists, accession numbers or GenBank taxon IDs. Specifically, Get GB allows the user to download GenBank data from a text list of accession numbers, allowing the user to select from multiple output formats depending on downstream analyses (GenBank, FASTA or phytab formats). We also created tools capable of downloading phylogenies and corresponding datasets from the PhyLoTA database (http://phylota.net), using a list of species or taxon ID for a group of interest. Trees with target species, FASTA and phytab format genetic data are saved as output, which can then be analyzed using other tools in Osiris.

#### Assembly and quality control of EST and Next-generation sequence data (Bioinformatics tools in Galaxy)

A primary focus of Galaxy itself is on analyzing high throughput genomic data, such that with Osiris tools installed, phylogeneticists can immediately leverage existing assembly tools (e.g. iAssembler, Trinity, Newbler, SOAPdenovo, Abyss, MIRA). After assembly, a critical next step is quality control. Galaxy already has wrappers for a variety of high throughput quality control (QC) scripts, focusing especially on Illumina FASTQ formats. These QC scripts combine data visualization and statistical analyses (for example identifying over represented sequence motifs that could indicate contamination by adapters or linkers), to generate reports of multiple QC steps simultaneously.

#### Determination of orthologous genes (Orthologs repository)

In order to provide the ability to partition genomes into orthologous genes for a given group of taxa, we created wrappers for the software package EvolMap [13]. Using a gene based clustering method informed by species relationships, EvolMap infers shared genes and gene families for a given set of genomes. This allows users to input genomes of their own choosing in order to target a specific group of taxa for ortholog selection. We then created wrappers for HmmBuild, HmmSearch and HaMStR [14]. Thus, results from EvolMap (or any other alignment) can be used to create hmms using HmmBuild. Then, using HmmSearch and/or HaMStR, the user can scan query sequences against a set of hmms. The resulting data can be aligned and concatenated for phylogenetic analyses. Incorporating these tools into workflows through the Galaxy platform is particularly useful, as the user can input virtually any FASTA format file (nucleotide or protein) as query, and subsequently combine all resulting ortholog hits.

#### Multiple Sequence Alignment and Concatenation (Alignment repository)

For the purpose of accelerated multiple sequence alignment, we created wrappers capable of taking both our new phytab and FASTA format input files for MUSCLE [17] and MAFFT [20] and PRANK [18]. As such, an entire multi-gene data set maintained in phytab format can be passed to a sequence alignment tool, and each gene aligned separately. Subsequently, the resulting alignments can be processed using Aliscore and Alicut [21], thereby identifying and removing ambiguous sections of the alignment in an objective manner, prior to phylogenetic analysis. All the genes stay together in a single phytab file, and the aligned genes can then be concatenated together or analyzed separately.

**Figure 1:**
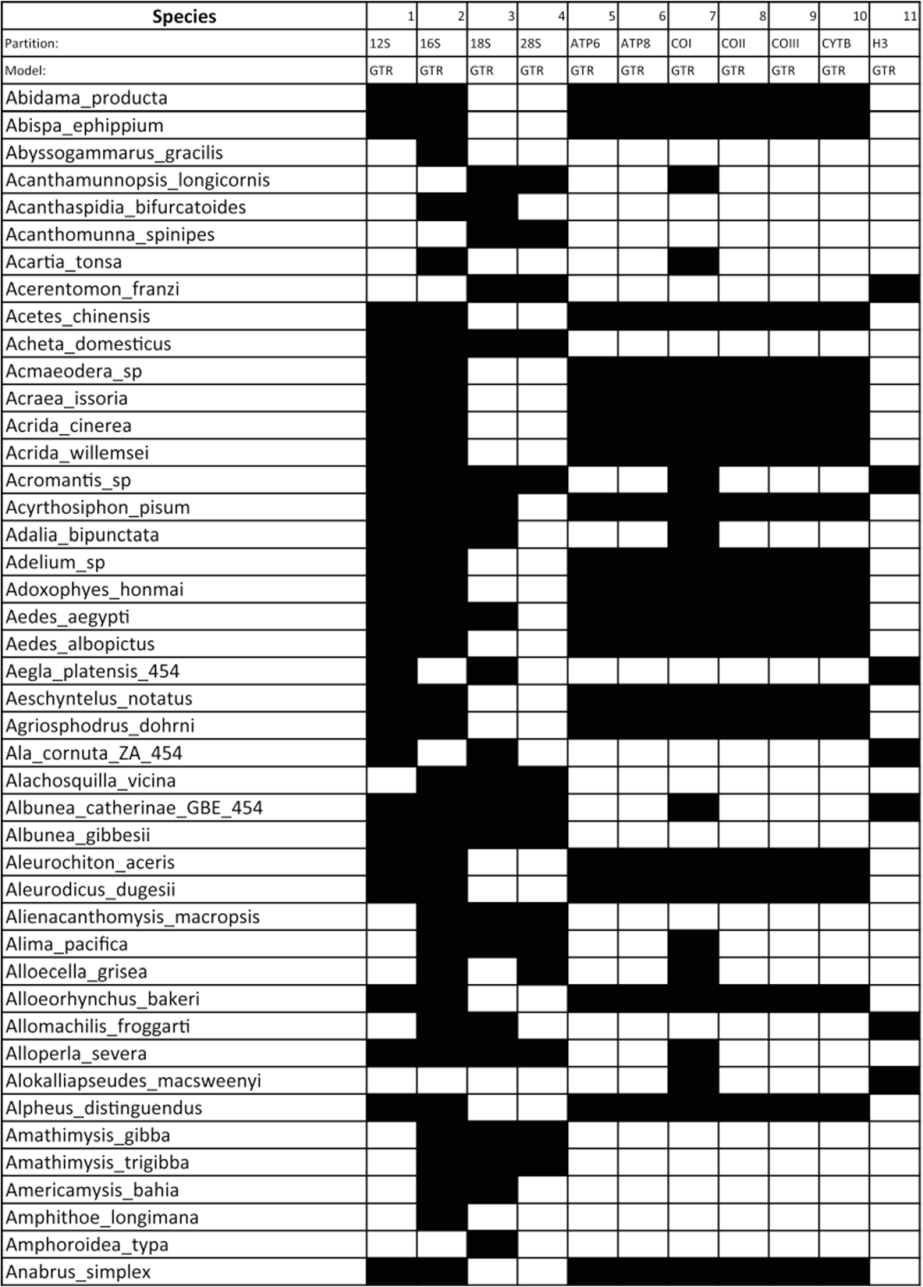
**Phylocatenator matrix.** Part of the output from Phylocatenator is shown as an html table representing gene coverage across species. The table contains the gene name, the model assigned to each partition (if that information is provided by the user prior to while running Phylocatenator), and the presence (black) or absence (white) of the gene for each taxon.

Currently, a complex step for phylogenetic analyses is concatenating together data sets with different taxonomic representation. Based on our experiences with multi-gene datasets, we have developed a script for Osiris called Phylocatenator, capable of taking a phytab format input file containing multiple genes (or morphological data) with uneven data coverage per species (Figure 1). The tool can filter data based on user-provided cutoff variables, such as minimum genes per species, minimum length of an aligned gene, and minimum species per gene. Furthermore, data can be filtered using a species list to select specific taxa for analyses, and the user can provide a table of models for each gene/partition, creating a partition file for use with RAxML [25]. Phylocatenator also outputs a file with a species list, names and lengths of genes/gene families, and an html table representing gene coverage across species (Figure 1). When combined with Galaxy’s workflow system, this tool easily allows for sophisticated and detailed exploration of the impacts of missing data and taxon sampling on resulting phylogenetics [32].

Because Phylocatenator uses our phytab format as input, and users may need to concatenate files which are in different formats, we also created an Osiris/Galaxy wrapper for FASconCAT [23], which can concatenate Phylip, Clustal and/or FASTA input files, and output FASTA, Phylip and/or Nexus for use in multiple possible downstream phylogenetics applications.

#### File Format Conversion (Phyloconversion repository)

A mundane and often time-consuming task is converting file formats for use in different computer packages. Galaxy itself already has a number of useful format conversion tools, including FASTA to tabular and tabular to FASTA. These tools make it easy for the user to switch between FASTA and phytab formats. Galaxy can also filter, sort and combine tabular file formats, making phylogenetic analyses with phytab files enormously flexible. For example, attributes such as rate of evolution of each gene partition can be estimated, and added as a separate column. The user could then sort on the rate of evolution column to retain only the slowest evolving genes for a phylogenetic analysis. This is just one example, and the flexibility is high enough that we expect analyses will be limited more by user imagination than by their computationally technical abilities.

#### Model-based phylogenetics (Phylogenies repository)

The heart of phylogenetic analysis is the estimation of the phylogenetic tree itself. Three mathematically and philosophically different approaches are common in the field: distance-, parsimony-, and model-based methods. In addition, philosophically different approaches to combining data partitions also exist, including concatenating data prior to tree estimation, and analyzing gene trees separately and estimating species trees from the gene trees [27,33-35]. We have already developed Osiris tools from each of these approaches. For parsimony, we have created a wrapper for RAxML to implement its parsimony search. For likelihood, we have implemented RAxML with a Galaxy interface very similar to the RAxML black box (http://phylobench.vital-it.ch/raxml-bb/). For Osiris, we have created different implementations of RAxML so that the user does not have to choose these. Specifically, we use an MPI version of RAxML for bootstrapping, and a Pthreads version for single-tree searches. This allows use of coarse- and fine-grained parallelization without the end user having to use command line arguments to send different types of jobs to a queue for different tasks. Galaxy can handle these different requests without the user knowing about it. As another ML implementation, GARLI exists on the Galaxy tool shed and it can be combined with our tools. We have made a simple wrapper for BEAST [1]. We currently have implemented one gene tree/species tree approach, NJst [36].

Model-based phylogenetic analyses often proceed first by statistical determination of the best-fit model of molecular evolution, given the data at hand. We have written wrappers for jModelTest [30] and ProtTest [37], which utilize phytab format, such that the user can more easily determine the best-fit models for many genes simultaneously. The output is a table with gene name in one column and best-fit model in another column, which can be passed to phylogenetic analysis programs downstream, to set the appropriate model separately for each data partition/gene.

#### Post-phylogeny visualization and analysis (Phylographics and Phylostatistics repositories)

Once a phylogenetic tree is estimated, there are many visualizations and analyses that can be conducted. For visualization of trees in Osiris, we use TreeVector from the Galaxy implementation of mothur (http://tinyurl.com/8zo558l), a Galaxy tool suite focused on microbial ecology. We also call R from Galaxy, and use the ape [38] library to generate phylogeny graphics. For example, we can produce separate trees for hundreds of gene families from one phytab file, and pass those results to an ape R script that produces a ‘book’ of tree graphics in a PDF file, one tree per page, that can be viewed to look for peculiarities, such as very long branches that could signal suspect raw data. We also have a tool to convert species names to GenBank taxon IDs, which can be passed to iTOL [39]. In addition to these existing tools, we propose to leverage iTOL [39] for automated annotation of clades on trees using GenBank taxonomy. Furthermore, we will continue to develop other post-phylogeny statistical tests. We have already implemented the SH test for comparing tree topologies [40] and we have a tool to calculate Phylogenetic Distances [41].

#### Phylogenetic Workflows in Galaxy

**Figure 2:**
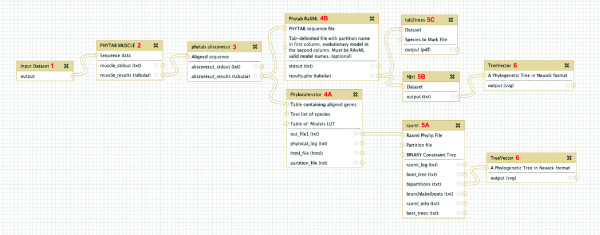
**Workflow.** Here we show a workflow constructed in Galaxy’s workflow editor. The analysis starts with the input dataset (1), which in this instance would be an unaligned tab-delimited, four-column phytab file. The raw phytab file then gets aligned (2) using phytab-MUSCLE, which will implement a multiple sequence alignment on each gene individually. After alignment, we implement a masking step (3) using phytab-aliscorecut, which will remove ambiguous regions separately from each gene. The data are now ready to be concatenated using phylocatenator (4A), and used to reconstruct a phylogeny with RAxML (5A). Alternatively or simultaneously, the data from step 3 can be used to estimate a separate phylogeny for each gene using phytab-RAxML (4B), which stores all gene trees in tabular format. Subsequently, gene trees can be used to estimate a species tree using NJst (5B), and/or all gene trees can be plotted individually for visual inspection using tab2trees (5C). Finally, any resulting tree file in Newick format can be plotted using TreeVector (6).

**Figure 3:**
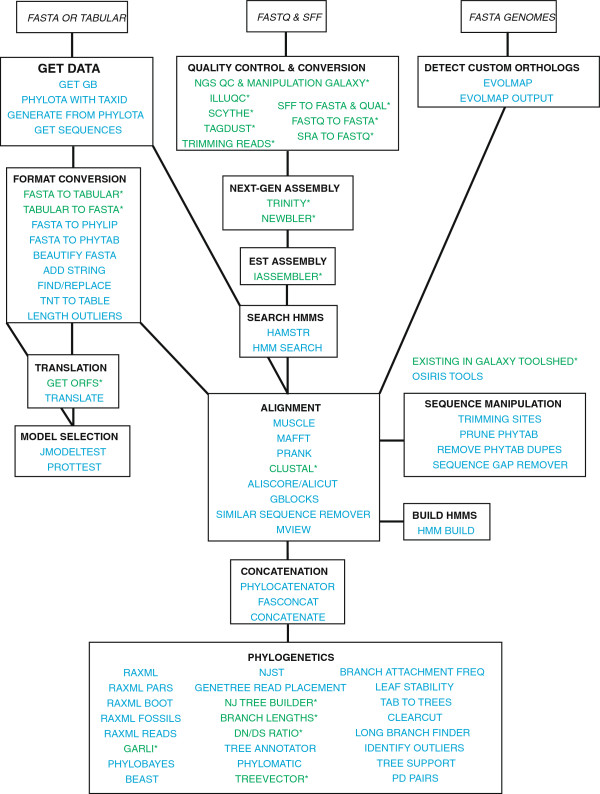
**All tools.** Here we show the various analyses Osiris in Galaxy is capable of. Different tools depicted in this figure can be combined to create complex phylogenetic workflows starting with a wide range of input files. Tools for which we created wrappers as part of this publication are in italics, while existing tools already available in Galaxy are denoted with an asterisk. Each box depicts an analysis category or different stage in a potential workflow. Lines connecting the boxes show different ways these tools can be combined based on input and output formats.

One of the clear advantages of using Osiris in Galaxy for complex phylogenetic analysis is the ability to combine multiple tools in a workflow in order to perform complex analyses at the click of a button. Within, Galaxy, sharable, reusable workflows are created using a GUI interface. One such example could include starting with raw unaligned sequence data, then proceeding with multiple sequence alignment and masking, followed by a partitioned and bootstrapped Maximum Likelihood analysis on a concatenated dataset, and/or a gene tree approach were phylogenies for each partition are combined to produce a species tree (Figure 2). This workflow is very flexible, and easily extended into more complicated analyses involving detection and removal of long branches, multiple phylogenetic analyses using model and distance based methods, divergence time estimation, and various post-tree analyses. Furthermore, the availability of tools we provide, along with the flexibility of the phytab format as input allows the user to combine data from a variety of sources, including FASTA, sff, FASTQ, SRA, GenBank, morphological data and a variety of others (Figure 3). This integration allows users to create large comprehensive datasets, drawing from multiple independent sources, in order to maximize species coverage while incorporating all available genetic data. Most importantly, it is the ease with which these workflows can be created that is key to the user-friendly nature of the Osiris platform in Galaxy.

## Conclusions

The diminishing cost of sequence data has transformed phylogenetic analysis, and studies examining hundreds or thousands of genes simultaneously are now commonplace. Recent methodological controversies in human genomics, which are at the forefront of bioinformatics analysis, should alert us to potential pitfalls caution [42]. As in any field, growing pains are inevitable, but it is essential that phylogenetics remain as transparent, replicable, reviewable, and accessible as possible. The Osiris platform in Galaxy helps to facilitate all of these goals.

Future development of Osiris will take three major directions: tool creation, research community involvement and increased computing power (cloud computing). As new phylogenetics programs are released, we will develop wrappers to include them in Osiris. This rapid ease of use, including a Galaxy tool called toolfactory that creates wrappers for existing scripts, will encourage users to incorporate the most current methods, whether in purely phylogenetic analyses or inter-disciplinary work. As more users join the Osiris/Galaxy community, they will share data, tools, and workflows that can be further developed by the community. Moreover, they can contribute their own tools. Finally, Galaxy is already prepared for changes in technological infrastructure [43,44], which will allow Osiris to move from local to cloud-based resources.

## Availability and requirements

**Project Name:** Osiris

**Project Home Page:**https://bitbucket.org/osiris_phylogenetics

**Project Demonstration Page:**http://galaxy-dev.cnsi.ucsb.edu/osiris/

**Operating System:** Any Internet Browser

**Programming Language:** Python, Perl, C, Java and others

**Other Requirements:** Install Galaxy (http://galaxyproject.org) and required tools

**License:** All original source code for Osiris tools is available under the MIT license (http://opensource.org/licenses/mit-license.html). **See below:**

The MIT License (MIT)

Copyright (c) 2013 Oakley et al. Permission is hereby granted, free of charge, to any person obtaining a copy of this software and associated documentation files (the "Software"), to deal in the Software without restriction, including without limitation the rights to use, copy, modify, merge, publish, distribute, sublicense, and/or sell copies of the Software, and to permit persons to whom the Software is furnished to do so, subject to the following conditions:

The above copyright notice and this permission notice shall be included in all copies or substantial portions of the Software.

THE SOFTWARE IS PROVIDED "AS IS", WITHOUT WARRANTY OF ANY KIND, EXPRESS OR IMPLIED, INCLUDING BUT NOT LIMITED TO THE WARRANTIES OF MERCHANTABILITY, FITNESS FOR A PARTICULAR PURPOSE AND NONINFRINGEMENT. IN NO EVENT SHALL THE AUTHORS OR COPYRIGHT HOLDERS BE LIABLE FOR ANY CLAIM, DAMAGES OR OTHER LIABILITY, WHETHER IN AN ACTION OF CONTRACT, TORT OR OTHERWISE, ARISING FROM, OUT OF OR IN CONNECTION WITH THE SOFTWARE OR THE USE OR OTHER DEALINGS IN THE SOFTWARE.

**Restrictions:** None

## Competing interests

The authors declare that they have no competing interests.

## Authors’ contributions

THO conceived phytab and other tools, coordinated project, wrote manuscript. MAA aided in tool development, project coordination and writing. RN wrote hamstr wrapper and other code. MSP aided in tool development and writing. CKCC aided in tool development and writing. WC aided in tool development. KBL wrote phytab and other code. All authors read and approved the final manuscript.
